# Misfolding of a Single Disulfide Bonded Globular Protein into a Low-Solubility Species Conformationally and Biophysically Distinct from the Native One

**DOI:** 10.3390/biom9060250

**Published:** 2019-06-25

**Authors:** Tomonori Saotome, Toshio Yamazaki, Yutaka Kuroda

**Affiliations:** 1 Department of Biotechnology and Life Science, Tokyo University of Agriculture and Technology (TUAT), Tokyo 184-8588, Japan; 2 NMR Facility, Division of Structural and Synthetic Biology, Center for Life Science Technologies, RIKEN, 1-7-22 Suehiro-cho, Tsurumi-ku, Yokohama City, Kanagawa 230-0045, Japan

**Keywords:** Single domain globular protein, single disulfide bond, protein refolding, protein misfolding, aggregation-prone species

## Abstract

In practice and despite Anfinsen’s dogma, the refolding of recombinant multiple SS-bonded proteins is famously difficult because misfolded species with non-native SS-bonds appear upon the oxidization of their cysteine residues. On the other hand, single SS-bond proteins are thought to be simple to refold because their cysteines have only one SS-bond partner. Here, we report that dengue 4 envelope protein domain 3 (DEN4 ED3), a single SS-bonded protein can be irreversibly trapped into a misfolded species through the formation of its sole intramolecular SS-bond. The misfolded species had a much lower solubility than the native one at pHs higher than about 7, and circular dichroism measurements clearly indicated that its secondary structure content was different from the native species. Furthermore, the peaks in the Heteronuclear Single Quantum Correlation spectroscopy (HSQC) spectrum of DEN4 ED3 from the supernatant fraction were sharp and well dispersed, reflecting the beta-sheeted native structure, whereas the spectrum of the precipitated fraction showed broad signals clustered near its center suggesting no or little structure and a strong tendency to aggregate. The two species had distinct biophysical properties and could interconvert into each other only by cleaving and reforming the SS-bond, strongly suggesting that they are topologically different. This phenomenon can potentially happen with any single SS-bonded protein, and our observation emphasizes the need for assessing the conformation and biophysical properties of bacterially produced therapeutic proteins in addition to their chemical purities.

## 1. Introduction

In practice, Anfinsen’s dogma [1] does not always apply well when refolding is performed in vitro, and many large multiple SS-bonded bacterially expressed proteins cannot fold correctly in test tubes [2,3]. A major problem is that incorrect SS-bonds are formed [4,5] resulting in misfolded aggregates [3,6,7,8]. In favorable cases, external factors such as chaperones that help avoid aggregation [9,10] and protein disulfide isomerase can reshuffle incorrect SS-bonds [11,12]. Similarly, solubility controlling peptide tags combined with low-temperature can stabilize the native state and help refold multiple SS-bonded proteins [13]. Nevertheless, refolding large multiple SS-bonded proteins in vitro remains challenging and optimizing such a protocol is difficult [14,15]. On the other hand, refolding single SS-bonded proteins is thought to be simpler because the cysteine residues have only one partner with which the SS-bond can form [16,17,18]. One thus expects that the cysteine residues can be air oxidized in the presence of a denaturant for maintaining the solubility of the proteins in the reduced unfolded state and remove the denaturant once air-oxidization of the cysteines is completed and the protein folded.

In this study, we investigated a low solubility species that appears as a precipitate during the refolding of dengue 4 envelope protein domain 3 (DEN4 ED3) from inclusion bodies [19]. We used DEN4 ED3 because it plays an important role in the viral neutralization process as it is recognized by the antibodies (two epitope regions are located in ED3) and the receptor binding site is located nearby ED3. ED3 has thus biological significant in terms of dengue’s viral propagation, survival, and infectivity. Furthermore, this is an immunoglobulin (Ig)-like fold which is found in several proteins (For example, Titin Ig (PDB ID: 1TIU)). Finally, ED3 is small, 106-residue, globular, beta-sheeted protein with a single intramolecular SS-bond (Figure S1). It can fold independently, and can be produced in large quantities which makes it an ideal model for biophysical studies. Because DEN4 ED3 is small and has only a single intramolecular SS bond, its refolding was anticipated to be simple. However, contrary to our expectations, almost half of the DEN4 ED3 precipitated at pH 8.0 when Guanidine Hydrochloride (GuHCl) was removed by dialysis during its refolding. Here we show that the precipitate, which is aggregation-prone and soluble only at low pH, has biophysical properties very different from the natively folded DEN4 ED3, which remained in the soluble fraction upon removal of GuHCl.

Importantly, the aggregation-prone species are not produced by intermolecular SS-bonds, as most molecules formed intramolecular bonds. Precipitation of misfolded proteins via the formation of non-native SS-bonds is commonly observed during the refolding of multiple SS-bonded proteins, but, to our knowledge, this is the first such report for a single SS-bonded protein. This phenomenon can potentially happen with any single SS-bonded protein, but it was unnoticed so far possibly because the precipitated fraction is usually discarded and not characterized.

## 2. Materials and Methods

### 2.1. Synthesis, Expression and Purification of the Protein

We used a previously constructed pET15b vector containing synthetic gene encoding the DEN4 ED3 sequence from UniProt (P09866; residues 575(294) to 679 (398)) at the endonuclease NdeI and BamHI sites [19]. The protein was overexpressed in Escherichia coli strain BL21 (DE3) pLysS as inclusion bodies in 1L of Luria Bertani (LB) medium at 37 °C. Protein expression was induced by the addition of isopropyl β-d-thiogalactopyranoside (IPTG) at the final concentration of 1 mM when the optical density at 590 nm of the culture was equal to 0.6. The cells were harvested 4 h after IPTG induction by mild centrifugation, and purified essentially as previously described [19].

The cells were lysed by sonication and the cysteines were air-oxidized for 36 h at 30 °C in 6 M GuHCl with 50 mM Tris-HCl (pH 8.7) buffer. The 6×histidine-tagged protein was purified by using nickel-nitrilotriacetic acid (Ni-NTA) (QIAGEN, Hilden, Germany) chromatography in the presence of 6 M GuHCl, followed by 15 h dialysis against Reverse Osmosis (RO) water (four times exchange of the outer solution) and 3 h dialysis against 10 mM Tris-HCl (pH 8.0) at 4 °C. After 20 min centrifugation at 8000 rpm and 4 °C, the precipitate was dissolved in 6M GuHCl supplemented with 10% acetic acid, and a second round of dialysis and centrifugation was performed as described above. The precipitate of the second dialysis was collected, dissolved in 6 M GuHCl with 10% acetic acid, dialyzed, and centrifuged for a third time.

Finally, the precipitate of the third dialysis was dissolved in 6M GuHCl with 10% acetic acid solution, followed by 15 h dialysis against RO water (four times exchange of the external solution) and 3 h dialysis against 20 mM Na-Acetate (pH 5.0) at 4 °C. The 6×histidine-tag was cleaved by Tobacco Etch Virus (TEV) protease-C9R [20] at the weight ratio of 1:3 (6×histidine-tagged TEV-protease C9R: 6×histidine-tagged DEN4 ED3), incubated at 30 °C for 24 h in the presence of 1 mM dithiothreitol (DTT). The sample was diluted 3-fold by 6M GuHCl with 50 mM Tris-HCl (pH 8.7), and the protein was further purified by a second passage through a Ni-NTA column. In the following experiments, we mainly analyzed the fraction harvested from the first supernatant (1st sup) and the one collected from the third precipitate (3rd ppt). The fractions in the first and second precipitates were recycled in the next round of dialysis after collecting minute aliquots for analysis. The protein’s identity was confirmed by Matrix Assisted Laser Desorption/Ionization-Time of Flight (MALDI-TOF) mass spectroscopy (TOF/TOF™ 5800, ABI SCIEX, Massachusetts, USA), and the molecular weights were within 10 daltons of the computed values.

### 2.2. Analytical Reverse Phase High Performance Liquid Chromatography

The proteins were analyzed by reverse phase high performance liquid chromatography (RP-HPLC) using a YMC-pack PROTEIN-RP column (4.6 mm × 25 mm column diameter, S = 5 µm) and absorbance at 220 nm was used to monitor the HPLC runs. Solution A (MilliQ-water + 0.1% trifluoroacetic acid (TFA)) and Solution B (Acetonitrile + 0.05% TFA) were used as a mobile phase with a flow rate of 1 mL/min and column temperature of 30 °C. An amount of 500 µL of the sample was prepared by mixing the protein solution with 10% (*v*/*v*) acetic acid, and by filtering the sample with a 0.20 μm membrane filter (Millex-GV; Millipore, Massachusetts, USA) to remove any large aggregates.

### 2.3. Circular Dichroism Measurements

Samples were prepared at a 0.2 mg/mL concentration in 50 mM sodium acetate buffer (pH 5.0) or Tris-HCl buffer (pH 8.0). The pH was adjusted by dialysis in the respective buffers, and the protein concentration was adjusted by diluting the protein solution with the buffer. The samples were filtered with a 0.20 μm membrane filter (Millex-GV, Millipore, USA) to remove aggregates. The concentrations and pHs of the samples were confirmed just before performing the experiments. Circular dichroism (CD) measurements were conducted on a J-820 spectropolarimeter (JASCO, Tokyo, Japan) using a 2 mm optical path length quartz cuvette. The sample’s temperature was increased from 20 °C to 90 °C with 10 °C increments and decreased back to 20 °C to check the reversibility of the temperature denaturation.

### 2.4. Dynamic Light Scattering Measurements

Samples for dynamic light scattering (DLS) were prepared in the same way as for CD at a protein concentration of 0.2 mg/mL. These measurements were performed by using 100 µL of the sample and a plastic cuvette with a Zeta-nanosizer Nano S (Malvern, Surrey, UK). The sample’s temperature was kept at 20 °C, and the hydrodynamic radius (*R*_h_) was calculated from size-volume graphs by using the Stokes-Einstein equation.

### 2.5. Mass Spectrometry (MALDI-TOF MS) Measurements

Matrix Assisted Laser Desorption/Ionization-Time of Flight (MALDI-TOF) MS measurements were performed by using the plate with the AB SCIEX TOF/TOF™ 5800 System (AB SCIEX, USA). The matrix solution was prepared by dissolving 10 mg of sinapic acid in 1 mL of a solution containing 50% acetonitrile and 0.1% trifluoroacetic acid. Samples for MALDI-TOF MS were prepared by mixing 1 µL of protein solution with 4 µL of matrix solution. One microliter (1 µL) of the sample mixtures were spotted and air-dried on the MALDI-TOF MS plate.

### 2.6. SDS-PAGE Measurements

Samples for sodium dodecyl sulfate polyacrylamide gel electrophoresis (SDS-PAGE) were prepared just after each of the three dialysis steps. The SDS-PAGE samples were as follows: An 8.8 µL aliquot from 17.5 mL of the first dialysis; a 5 µL aliquot from 10 mL of the second dialysis; and a 2 µL aliquot from 4 mL of the third dialysis. The aliquots were centrifuged 20 min at 8000 rpm and 4 °C, and the supernatants were separated from the precipitates, and the precipitates were dissolved in MilliQ in the same volume as the supernatants.

Next, each protein solution was mixed with sample buffer at equal volumetric ratio. The sample buffer was prepared by mixing with 12 mL of 0.5 M Tris-HCl (pH 6.8), 24 mL of 10% SDS solution, 12 mL of glycerol, 24 mL of Milli-Q water, and one spoonful of Bromophenol Blue. The sample buffer was mixed with 2-mercaptoethanol or MilliQ at the volumetric ratio of 3:1 beforehand. The SDS-PAGE was performed by using 17.5% acrylamide gels, at 20 mA as constant current for 90 min. The gels were stained using Coomassie Brilliant Blue (CBB) solution.

### 2.7. Tryptophan and ANS Fluorescence Measurements

Samples for tryptophan fluorescence measurements were prepared in the same way as for CD measurements at a protein concentration of 0.2 mg/mL. Samples for 8-Anilinonaphthalene-1-sulfonic acid (ANS) fluorescence measurements were prepared by mixing 0.2 mg/mL protein solution with 1 mM ANS at equal volumetric ratio and incubated at 20 °C for 5 min in the dark. The samples were filtered with a 0.20 μm membrane filter to remove aggregates. The concentrations and pHs of the samples were confirmed just before performing the experiments.

The fluorescence measurements were performed by using 200 µL of the sample and a quartz cuvette with a FP-8500 (JASCO, Tokyo, Japan) fluoro-spectrometer. The sample’s temperature was kept at 20 °C, and the emission at 345 nm was monitored with excitation at 295 nm for tryptophan fluorescence, and the emission at 480 nm was monitored with excitation at 380 nm for ANS fluorescence.

### 2.8. Nuclear Magnetic Resonance Measurements

Samples for nuclear magnetic resonance (NMR) measurements were prepared at a ^15^N-labelled protein concentration of 0.2 mg/mL in 50 mM sodium acetate buffer (pH 5.0). The samples were centrifuged at 20,000× *g* for 5 min and at 4 °C to remove aggregates. The concentrations and pHs of the samples were confirmed just before performing the experiments, and 20 µL of D_2_O was added to 300 µL of the samples for deuterium lock. All NMR experiments were performed at 25 °C on a Bruker BioSpin ^1^H-600-MHz AVANCE600 NMR spectrometer. The two-dimensional (2D)^1^H-^15^N Heteronuclear Single Quantum Correlation spectroscopy (HSQC) spectra were acquired with spectral widths of 9615 Hz (16.02 ppm) and 6080 Hz (100.0 ppm), respectively, in the ^1^H and ^15^N dimension. A total of 2048 complex points were collected in the ^1^H dimension and 256 complex points in the ^15^N dimension. The data were zero-filled to 4 k × 512 points, and a 90-degree shifted sine-bell window was applied before Fourier transformation. No baseline correction was applied. The water signal was suppressed by polynomial fitting in the ^1^H time domain, and a baseline correction in the f2 dimension.

### 2.9. Refolding of the DEN4 ED3-3rd Precipitation (ppt) by Breakage and Reformation of the SS-Bond

We dissolved 6 mg of DEN4 ED3-3rd ppt in 1 mL of 6 M GuHCl with 50 mM Tris-HCl (pH 8.7) buffer containing 100 mM DTT, and incubated at 4 °C for 1 h to cleave the SS-bond. Thereafter, acetic acid at a final concentration of 10% was added to inhibit both air oxidation and decrease the redox potential of DTT by lowering the pH. The sample was dialyzed against RO water (four times exchange of the outer solution) at 4 °C for 15 h to remove the DTT from the sample. The dialyzed sample was then transferred into a sample tube, and 3 mL of 6 M GuHCl with 50 mM Tris-HCl (pH 8.7) buffer was added to the sample, which underwent air-oxidation at 30 °C for 36 h. Finally, the sample was dialyzed against 10 mM Tris-HCl water at 4 °C for 15 h to remove any trace of GuHCl from the sample.

## 3. Results and Discussion

### 3.1. DEN4 ED3 in the Supernatant (sup) and the Precipitate (ppt) Are Chemically Identical

In order to fully separate the pellet from the supernatant, we performed three rounds of dialysis, where the pellet from the previous round was dissolved in 6 M GuHCl, which was removed by dialysis and the supernatant was again separated from the pellet. The supernatants of each round of dialysis (DEN4 ED3-1st sup, DEN4 ED3-2nd sup, DEN4 ED3-3rd sup), and the precipitate of the final round of dialysis (DEN4 ED3-3rd ppt) were recovered.

MALDI-TOF MS measurements indicated that the molecular mass of both DEN4 ED3-1st sup and DEN4 ED3-3rd ppt were identical to the computed value within an experimental error of 10 daltons to the value computed from DEN4 ED3 sequence (Figure S2, Table S1). This indicated that the two species were chemically identical. Similarly, SDS-PAGE of DEN4 ED3-1st sup and DEN4 ED3-3rd ppt in the presence of reductant indicated a single band at 11 kDa.

### 3.2. The Conformation of DEN4 ED3 in the Supernatant and the Precipitate Are Distinct

The protein concentration of DEN4 ED3-2nd sup and 3rd sup were approximately 0.20 mg/mL in contrast to 1.1 mg/mL for DEN4 ED3-1st sup (Table S2), which contained only the natively folded species as assessed by CD measurements (Figure S3). This suggested that the solubility of the misfolded DEN4 ED3 is one-fifth of the natively folded DEN4 ED3 at pH 8.0. The CD spectra of DEN4 ED3-2nd sup and 3rd sup could be essentially reconstructed by a linear combination of the spectra of DEN4 ED3-1st sup and 3rd ppt, indicating that the fractions contain a mixture of folded and misfolded DEN4 ED3.

**Figure 1:**
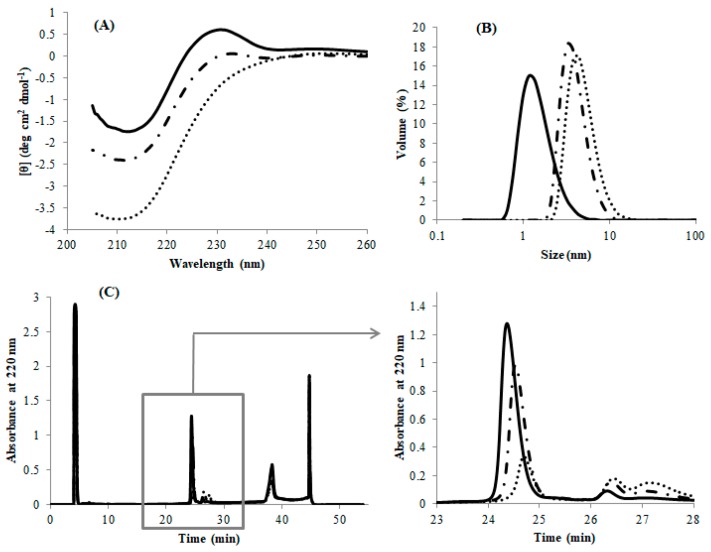
The physicochemical characterization of DEN4 ED3-1st sup (solid line); DEN4 ED3-2nd sup (dotted dashed line); DEN4 ED3-3rd sup (dotted line) at the protein concentration of 20 µM. (**A**) Circular dichroism (CD) spectra at pH 8 and 20 °C. (**B**) Size-volume graphs measured by dynamic light scattering (DLS) at pH 8 and 20 °C. (**C**) Reverse phase high performance liquid chromatography (RP-HPLC) chromatograms at pH 8 and 30 °C. The flow rate was kept as 1 mL/min.

Circular dichroism indicated that DEN4 ED3-1st sup was folded into a beta-sheet structure as reported in previous studies [21,22] (Figure 1A), and the CD spectra remained unchanged between pH 5.0 and 8.0 (Figure S3). On the other hand, the CD spectrum of DEN4 ED3-3rd ppt could be measured only at pH 5.0, where it was soluble, but the spectrum was substantially different both from that of DEN4 ED3-1st sup and of previously reported spectra [22,23]. In addition, DEN4 ED3-1st sup was native until 60 °C, and complete denaturation occurred at above 80 °C. On the other hand, no thermal denaturation occurred for DEN4 ED3-3rd ppt as monitored by CD even at 80 °C.

The hydrodynamic radius (*R*_h_), as measured by DLS, of DEN4 ED3-1st sup was smaller than 2 nm at both pH 5.0 and 8.0, whereas that of DEN4 ED3-3rd ppt was 7.91 nm at pH 5.0, and 747.2 nm at pH 8.0 (the sample was white and cloudy) (Figure S4, Table S3). This suggested that the misfolded DEN4 ED3 is oligomeric even though it remains in the supernatant at acidic pH. The *R*_h_ of DEN4 ED3-2nd sup and DEN4 ED3-3rd sup at pH 5 were 4.22 nm and 5.24 nm, respectively, and larger than the *R*_h_ of DEN4 ED3-1st sup (1.57 nm) (Figure 1B) suggesting that some misfolded proteins were mixed.

Finally, RP-HPLC of DEN4 ED3-1st sup (Figure 1C) exhibited an intense, well-shaped, single peak eluting at 24.5 min, as reported in our previous studies with the native DEN4 ED3 at a protein concentration of 0.2 mg/mL. On the other hand, DEN4 ED3-3rd sup at the very same concentration of 0.2 mg/mL exhibited a weak and broad peak with an elution time of 26.5 min. Altogether, these observations indicated that two species with different conformation and biophysical properties were present, and that one can completely separate the two species by multiple rounds of pH precipitation.

### 3.3. Further Structural and Biophysical Properties of the Misfolded DEN4 ED3

**Figure 2:**
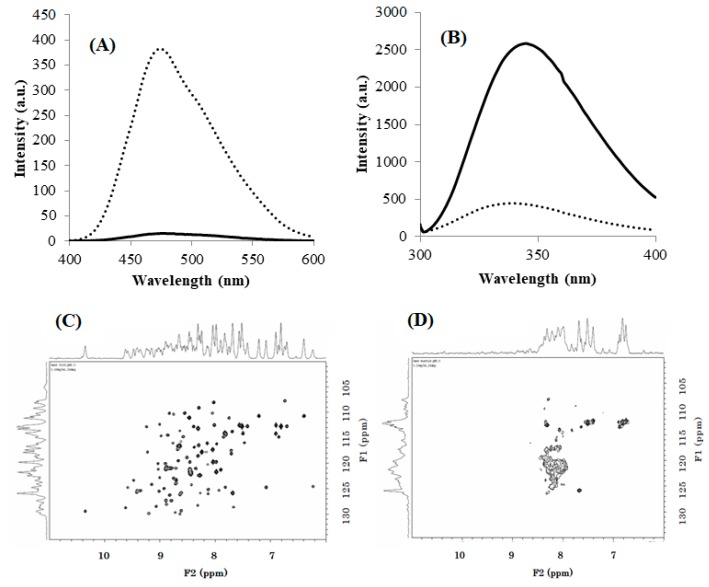
(**A**) 8-Anilinonaphthalene-1-sulfonic acid (ANS) fluorescence spectra and (**B**) Tryptophan fluorescence spectra of DEN4 ED3-1st sup (solid line) and DEN4 ED3-3rd ppt (dotted line) at pH 5.0 and 20 °C (**C**), (**D**): HSQC spectra of 15N-labelled DEN4 ED3-1st sup (**C**) and DEN4 ED3-3rd ppt (**D**) at 0.2 mg/mL, pH 5.0 and 25 °C.

We further analyzed the biophysical properties of the misfolded species using various spectroscopic methods. First, we measured the ANS and tryptophan fluorescence in order to determine the physicochemical properties of the molecular surface. ANS is an amphiphilic molecule that binds to partially globular denatured proteins (molten globule state [23,24,25]), but not to wholly denatured nor to natively folded proteins. The ANS fluorescence of DEN4 ED3-3rd ppt was over 25 times stronger than that of DEN4 ED3-1st sup (Figure 2A), strongly suggesting that DEN4 ED3-3rd ppt was partially denatured and probably in a molten globule-like state [26,27].

Next, the intensity and emission wavelength of tryptophan fluorescence is a criterion for protein folding/unfolding because upon unfolding water molecules will quench the fluorescence of tryptophans that become exposed but are buried in the hydrophobic core of the native protein and thus protected from water molecules. The tryptophan fluorescence of DEN4 ED3-1st sup was five time stronger than that of DEN4 ED3-3rd ppt (Figure 2B), and red shifted by 6 nm. This again suggested that DEN4 ED3-1st sup was natively folded, but that the tryptophan in DEN4 ED3-3rd ppt was accessible to water molecules as it would happen in a molten globule state [28,29,30].

Finally, NMR experiments indicated sharp and well-dispersed cross peaks in the HSQC spectrum of DEN4 ED3-1st sup (Figure 2C), whereas a few broad, barely observable peaks appeared near the spectrum’s center of DEN4 ED3-3rd ppt (Figure 2D), at around ^1^H = 8 ppm/^15^N = 120 ppm indicating that most amino acid residues didn’t retain a specific rigid tertiary structure [28,31,32,33]. Indeed, the chemical shifts of proteins oligomerized keeping a natively folded structure would remain close to their native values, and the peaks would just broaden [31,33]. So, it is reasonable to interpret the HSQC spectrum of the misfolded DEN4 ED3-3^rd^ ppt as that of a protein that is both unfolded (average chemical shift) and aggregated (broadening).

**Figure 3:**
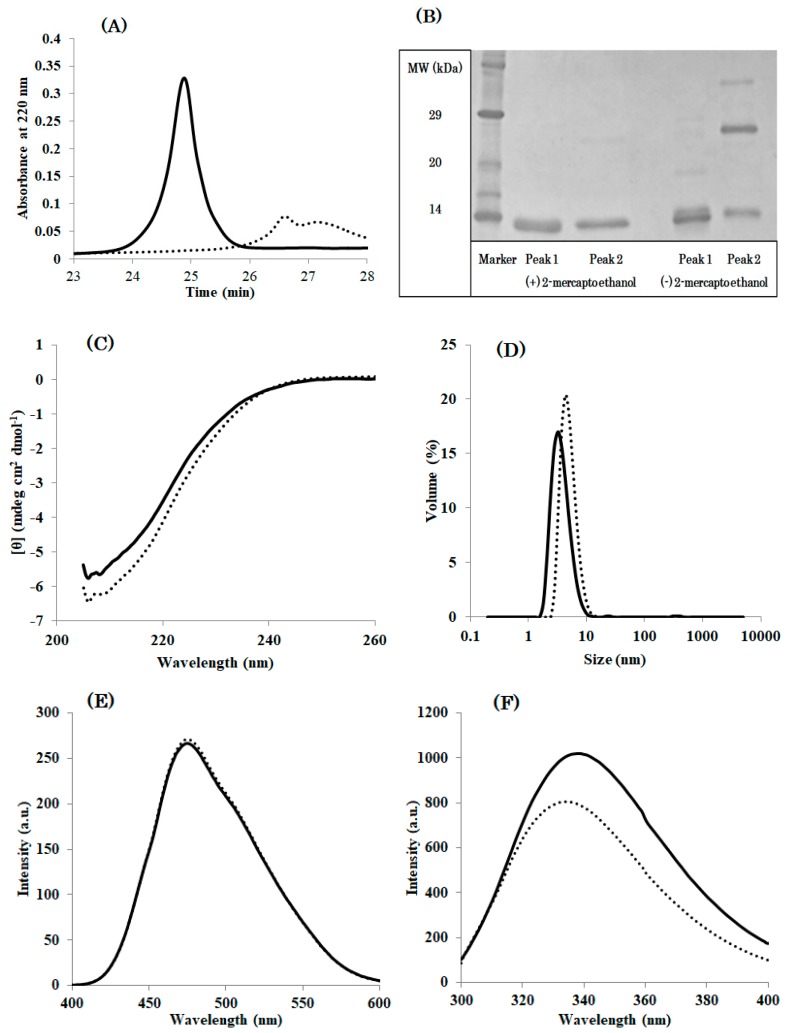
Further examination of Peak 1 (solid line) and Peak 2 (dotted line) obtained at RP-HPLC of DEN4 ED3-3rd ppt. (**A**) RP-HPLC chromatograms of two peaks. (**B**) SDS-PAGE result of two peaks in the presence or absence of 2-mercatptoethanol. (**C**) CD spectra at pH 5 and 20 °C. (**D**) Size-volume graphs measured by DLS at pH 5 and 20 °C. (**E**): ANS fluorescence spectra at pH 5.0 and 20 °C. (**F**) Tryptophan fluorescence spectra at pH 5.0 and 20 °C.

Additionally, DEN4 ED3-3rd ppt showed two peaks at RP-HPLC (Figure 3A). SDS-PAGE implied peak 1 is derived from a misfolded monomer although peak 2 contained both a misfolded monomer and disulfide bounded dimer (Figure 3B). This oligomeric species would be a dimer with intermolecular disulfide bond. However, misfolding and aggregation induced by intermolecular non-native SS bonds is a common phenomenon. On the contrary, the misfolding of monomeric proteins with a single intramolecular SS bond is surprising and highly novel. We thus focused on the misfolded monomeric species rather than the oligomeric species. and we show that a misfolded species with a single intramolecular SS bond can be purified by RP-HPLC (Figure 3). Moreover, CD, ANS, Tryptophan (Trp) fluorescence, and DLS indicated that the monomeric species with a single intramolecular SS bond possess physicochemical properties completely different from that of the natively folded protein (Figure 3C–F, Table S4). In particular, ANS and Trp fluorescence suggested that the misfolded proteins had molten globule like properties (Figure 3E,F). However, in sharp contrast with the molten globule state [34,35], the misfolded species is not in equilibrium with the native state: once the single intramolecular SS bond is formed it cannot interconvert into the native state. Thus, the misfolded species that is observed here is an off-pathway species, which is again distinct from the molten globule state that is thought to be a folding intermediate leading to the formation of a native state [36,37,38].

### 3.4. Conversion of the Low Solubility Misfolded Species into the Native One by the Reformation of SS-Bond

**Figure 4:**
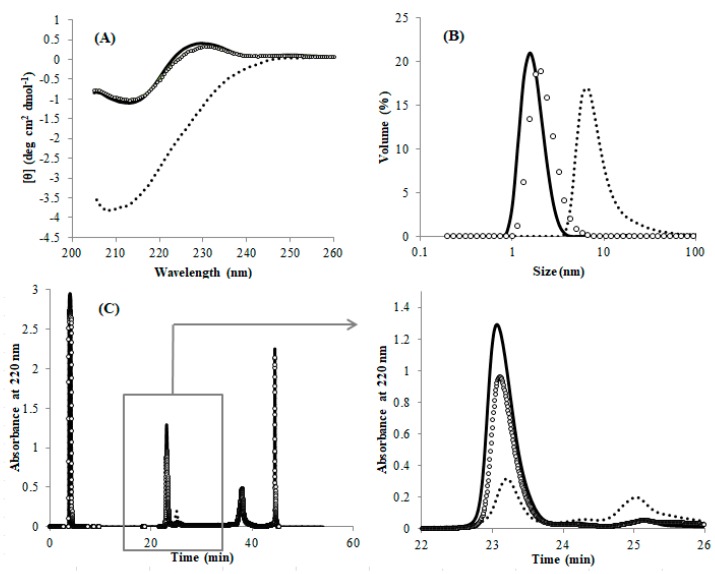
Physical properties of DEN4 ED3-1st sup (solid line); DEN4 ED3-3rd ppt (dotted line); DEN4 ED3-3rd ppt after the incubation with 100 mM DTT (white circle) at a protein concentration of 20 µM. (**A**) CD spectra at pH 5 and 20 °C. (**B**) Size-volume graphs measured by DLS at pH 8 and 20 °C. (**C**) RP-HPLC chromatograms at pH 8, 30 °C and a flow rate of 1 mL/min.

As described above, the misfolded DEN4 ED3 cannot be solubilized and thus refolded by merely dissolving the precipitate in GuHCl and removing it (Figure S1, Table S2). However, we observed that the misfolded DEN4 ED3 can be converted to the natively folded DEN4 ED3 by cleaving the SS-bond and re-forming it. Namely, we first cleaved the SS-bond of DEN4 ED3-3rd ppt by incubation at 4 °C for 1 h with 100 mM DTT. After removing DTT, the samples were air oxidized again in GuHCl. All of the experimental data (CD, DLS, and RP-HPLC; Figure 4) of DEN4 ED3-3rd ppt measured after the re-formation of the SS-bond were close, if not identical to that of DEN4 ED3-1st sup.

Altogether, these results show that the two fractions represent two species that are chemically identical and can interconvert into each other only by cleaving and reforming the intramolecular SS-bond: the protein is trapped into one of the species once the SS-bond is formed.

Finally, the misfolded low solubility species can potentially occur in any single SS-bonded globular protein, but it was probably unnoticed so far because the inclusion body fraction is usually discarded and not characterized; or when the protein is refolded from the inclusion body or the precipitated fraction, one usually reduces the cysteins while solubilizing the protein, thus cleaving the SS bond and enabling the formation of the native species.

## 4. Conclusions

In this study, we thoroughly characterized the fraction of DEN4 ED3 that precipitated at neutral pH during its purification. This fraction is usually discarded, and only proteins in the supernatant are analyzed. Proteins in the two fractions were chemically equivalent but possessed different biophysical properties such as secondary and tertiary structure, particle size, and solubility. The RP-HPLC peaks of the two species were distinct and apart from each other, but the simplest way to isolate them was precipitation at pHs higher than pH 7. The supernatant fraction at neutral pH contained only the correctly folded native ED3, and the precipitated fraction contained the misfolded species. Importantly, the misfolded and natively folded species could interconvert into each other only by breaking the SS-bonds and reforming them. Whether this finding is of general significance or specific to ED3 will need investigation with different proteins, however such a phenomenon is unexpected and unique and is worth being thoroughly documented. Furthermore, this finding suggests the importance of assessing, in addition to their chemical purity, the conformations and biophysical properties of bacterially produced proteins, even if they are small, single SS-bonded globular proteins.

## Supplementary Materials

The following are available online at https://www.mdpi.com/2218-273X/9/6/250/s1. Tables S1–S4 and Figures S1–S4.
