# Supplementary material for: Misfolding of a Single Disulfide Bonded Globular Protein into a Low-Solubility Species Conformationally and Biophysically Distinct from the Native One
Source: Biomolecules. 2019 Jun 25;9(6):250. doi: 10.3390/biom9060250 (PMC6627273; doi:10.3390/biom9060250)
Supplement: Supplementary file 1 [file biomolecules-09-00250-s001.pdf]

Supporting Information

Misfolding of a single disulfide bonded globular protein into a low-solubility species conformationally and biophysically distinct from the native one

Tomonori Saotome[a], Toshio Yamazaki[b], Yutaka Kuroda[a]

[a] Department of Biotechnology and Life Science, Tokyo University of Agriculture and Technology, Tokyo 184-8588, Japan
[b] NMR Facility, Division of Structural and Synthetic Biology, Center for Life Science Technologies, RIKEN, 1-7-22 Suehiro-cho, Tsurumi-ku, Yokohama City, Kanagawa 230-0045, Japan

Name	Est. Mw (Da)	Cal. Mw (Da)	Error (%)
DEN4 ED3-1st sup	11376.01	11383.06	6.19×10^{-2}
DEN4 ED3-3rd ppt	11376.01	11386.62	9.32×10^{-2}

Table S1. The estimated molecular weight (Est. Mw) by MALDI-MS and calculated molecular weight (Cal. Mw) by Protparam in DEN4 ED3-1st sup and 3rd ppt. The error was calculated by using the following formula (|Est. Mw –Cal. Mw| / Est. Mw ×100).

Name	Concentration (mg/ml)	Volume (ml)	Estimated yield (mg)	A_{260}/A_{280}
1st sup	1.08	15	16.2	0.65
1st ppt	2.16	4	8.64	0.73
2nd sup	0.29	6	1.74	0.70
2nd ppt	1.94	3	5.82	0.73
3rd sup	0.17	6	1.02	0.86
3rd ppt	2.52	2	5.04	0.74

Table S2. The yield of DEN4 ED3 in the supernatant (sup) and precipitation (ppt) after dialysis, three times. The protein concentrations were measured by NanoDrop 2000 using an extinction coefficient of 280 nm. The supernatant fractions were measured as they were, and the precipitated fractions were measured by dissolving in 6 M GuHCl with 10% acetic acid (pH 2.7).

	DEN4 ED3-1st sup	DEN4 ED3-3rd ppt
pH 5.0	1.97	7.91
pH 8.0	1.61	747.2

Table S3. Hydrodynamic radius (nm, R_h) of DEN4 ED3-1st sup and 3rd ppt at pH 5.0 and 8.0 after the cleavage of a 6×histidine-tag by TEV protease-C9R. The R_h values were calculated from DLS size-volume graphs.

Peak 1	Peak 2
3.78	5.04

Table S4. Hydrodynamic radius (nm, R_h) of Peak 1 and Peak 2 obtained at RP-HPLC of DEN4 ED3-3rd ppt at pH 5.0 and 20°C with a 6×histidine-tag. The R_h values were calculated from DLS size-volume graphs.

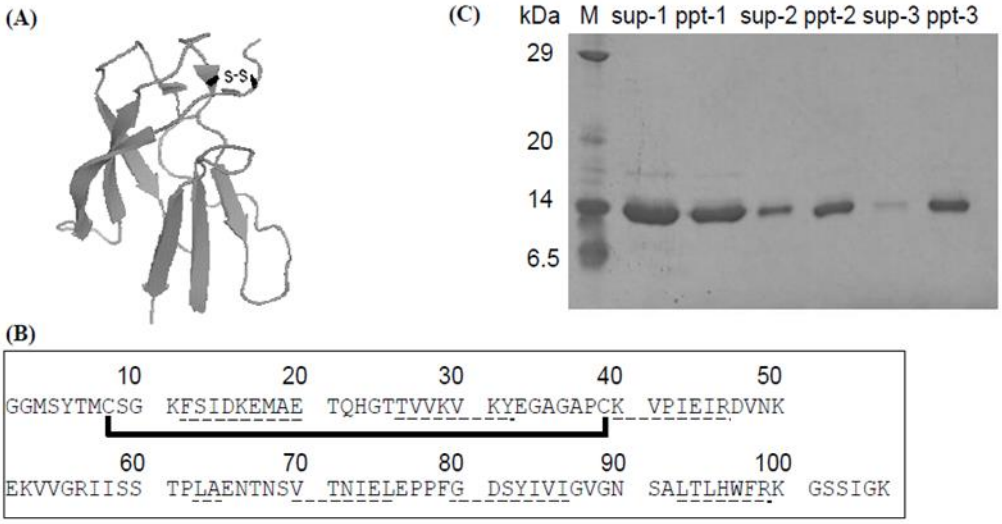

Figure S1: Structure, sequence, and SDS-PAGE of DEN4 ED3. (A) Ribbon model of DEN4 ED3 is drawn using Pymol and the PDB structure 3WE1. The SS bond is shown with “SS”. (B) Sequence of DEN4 ED3. The SS-bond is shown with a continuous black line, and the residues forming the b-strand are underlined. (C) SDS page of DEN4-Ed3 during its purification. Usually, DEN4 ED3 is purified from the first supernatant. After two additional rounds of solubilization and precipitation, all of the protein remained in the precipitate (ppt3), which we analyzed in this study. M: Protein marker; sup-1: DEN4 ED3-1st sup; ppt-1: DEN4 ED3-1st ppt; sup-2: DEN4 ED3-2nd sup; ppt-2: DEN4 ED3-2nd ppt; sup-3: DEN4 ED3-3rd sup; ppt-3: DEN4 ED3-3rd ppt.

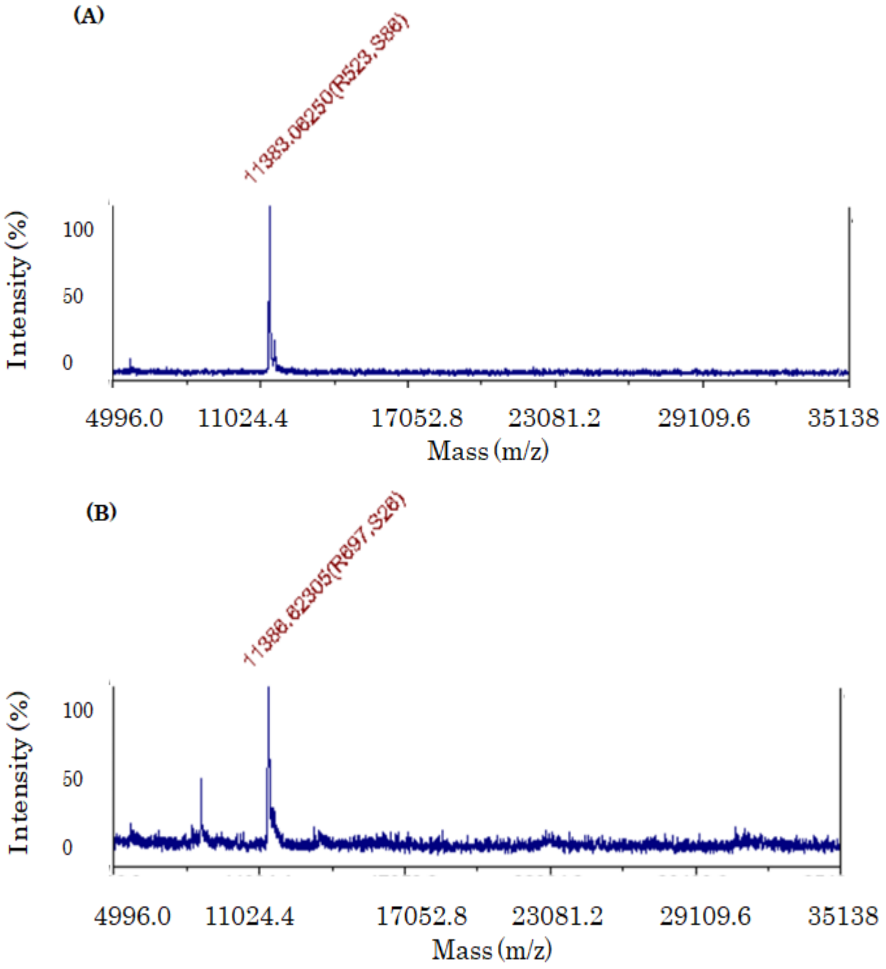

Figure S2. The MALDI-MS spectra of (A) DEN4 ED3-1st sup and (B) DEN4 ED3-3rd ppt at the protein concentration of 1 μM.

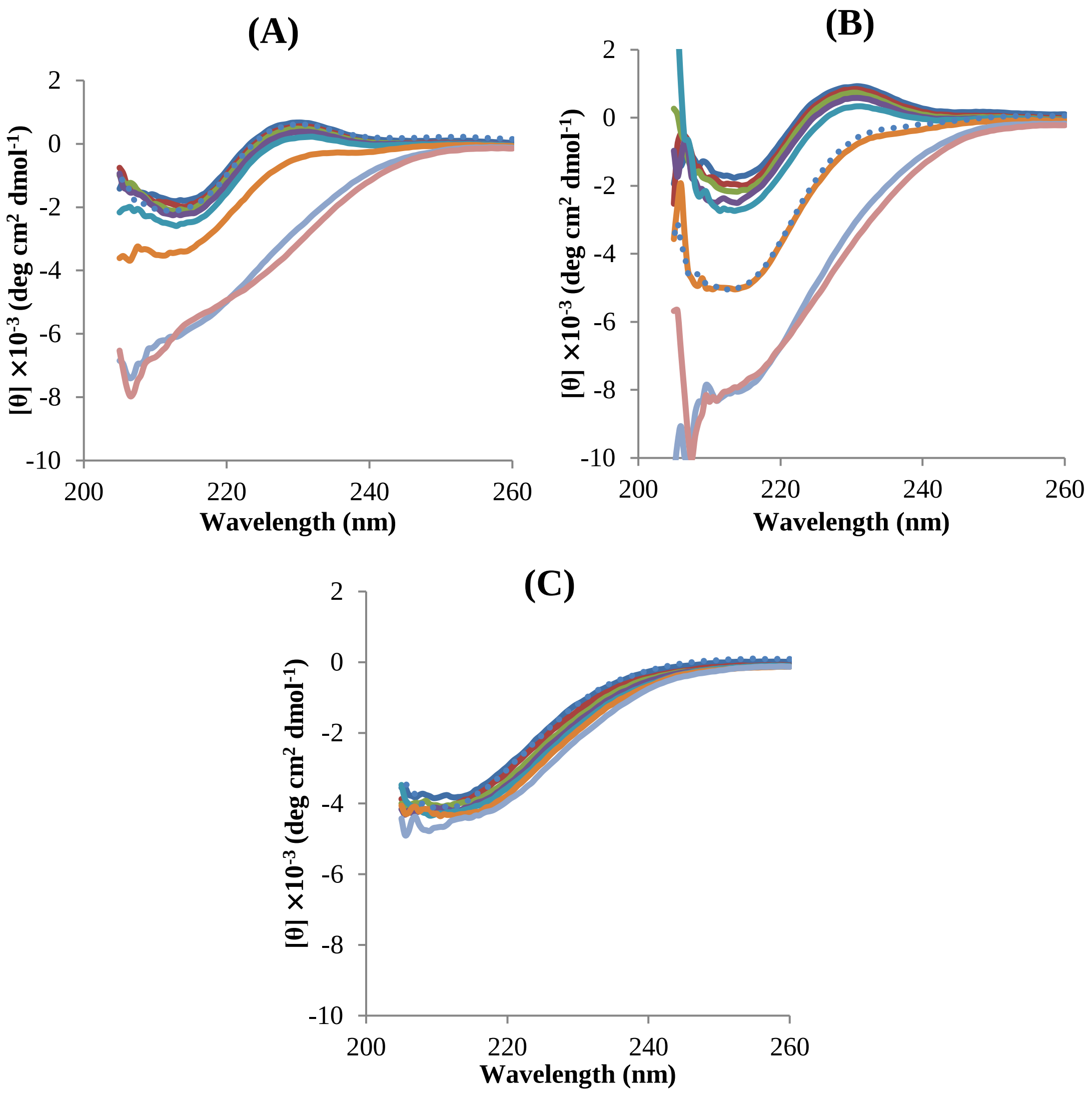

Figure S3. The temperature effect on CD spectra of (A) DEN4 ED3-1st sup (pH 5.0) (B) DEN4 ED3-1st sup (pH 8.0) (C) DEN4 ED3-3rd ppt (pH 5.0) at 0.2 mg/ml, between 20°C and 90°C. 20°C (Blue); 30°C (Red); 40°C (Green); 50°C (Purple); 60°C (Dark blue); 70°C (Orange); 80°C (Sky blue); 90°C (Pink); 20°C after heating (Blue dotted line).

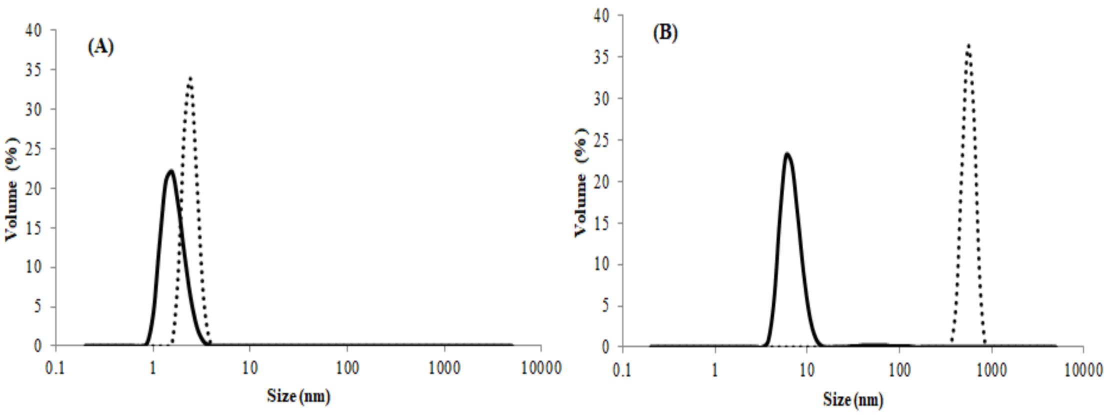

Figure S4. DLS size-volume graphs of (A) DEN4 ED3-1st sup; (B) DEN4 ED3-3rd ppt at pH 5.0 (solid line) and pH 8.0 (dotted line). The 6×histidine-tags attached to N-terminus of DEN4 ED3 were cleaved by TEV protease-C9R just before DLS measurements.
